# A Comparative Transcriptome Analysis between Wild and Albino Yellow Catfish (*Pelteobagrus fulvidraco*)

**DOI:** 10.1371/journal.pone.0131504

**Published:** 2015-06-26

**Authors:** Ming Zou, Xiaoting Zhang, Zechao Shi, Li Lin, Gang Ouyang, Guirong Zhang, Huan Zheng, Kaijian Wei, Wei Ji

**Affiliations:** 1 Department of Aquatic Animal Medicines, College of Fisheries, Huazhong Agricultural University, Wuhan, Hubei, 430070, China; 2 Key Laboratory of Freshwater Animal Breeding, Ministry of Agriculture, College of Fisheries, Huazhong Agricultural University, Wuhan, Hubei, 430070, China; 3 Freshwater Aquaculture Collaborative Innovation Centre of Hubei Province, Wuhan, Hubei, 430070, China; 4 Key Laboratory of Freshwater Biodiversity Conservation, Ministry of Agriculture, Yangtze River Fisheries Research Institute, Chinese Academy of Fishery Sciences, Wuhan, Hubei, 430223, China; 5 The Key Laboratory of Aquatic Biodiversity and Conservation, Institute of Hydrobiology, Chinese Academy of Sciences, Wuhan, Hubei, 430070, China; Chinese Academy of Fishery Sciences, CHINA

## Abstract

Body colours are important and striking features for individual survival and reproductive success, in particular in vertebrates where mating behaviour and mate preference may be strongly influenced by non-normal phenotypes. Pigmentation disorders may be generated by disruption of one or many independent genes as well as by environmental factors. The first discovery of albino yellow catfish (*Pelteobagrus fulvidraco* Richardson) with golden skin colour from fish farms in China provides us valuable material to study the molecular mechanism underlying the abnormalities of pigmentation. In this study, transcriptome sequencing of fin tissues corresponding to the distinct body colours, wild type and mutant albino yellow catfish, were performed using Illumina sequencing technology. Based on next-generation sequencing technology and *de novo* assembly, we generated a transcriptome of *P*. *fulvidraco*. A number of genes differentially expressed between the wild types and albinos were identified, suggesting their contribution to the different phenotypes and fitness. However, non-synonymous mutations result from single nucleotide substitutions residing in coding regions may not contribute to such differences. Based on the high-throughput expression data generated for the two different types of *P*. *fulvidraco*, we found that alterations of expression pattern may be more common than non-synonymous mutations. The transcriptome of *P*. *fulvidraco* will be an invaluable resource for subsequent comparative genomics and evolutionary analyses of this economically important fish.

## Introduction

Body colours and colour patterns are significant and striking phenotypic traits in vertebrates and have important functions such as contributing to mate selection, species recognition, crypsis, and warning or threatening of predators. Disorders of pigmentation can cause variation in body colour, among which albinism was reported as an inborn error of metabolism proposed by Garrod [1]. Albinism is characterized by a series of genetic abnormalities, which is caused by a deficiency of melanocytes or the malfunction of melanin production [2]. Albino individuals occur in a variety of vertebrates including mammals [3], reptiles [4], amphibians [5], birds [6] and fishes [7] and are usually accompanied by a series of pathology changes, such as anemia, osteopetrosis,reduced eyes,deafness,neurological and skeletal defects [8–10]. Albino individuals typically experience much higher levels of selection (i.e., phenotype-specific mortality and/or reduced reproductive fitness) in the natural environment than normal individuals and as a consequence albinos in the wild are usually rare. Pigment cells of vertebrates are derived from the dorsal neural crest during embryonic development. So far, at least seven types of pigment cells have been reported, including melanocytes (black or dark brown), xanthophores (yellow), erythrophores (red/orange), iridophores (reflective silver, blue, or gold), leucophores (white), cyanophores (blue) and irido-erythrophores (violet) [11, 12]. Only bony fishes possess all seven types of pigment cells [11]. These cells are concerned because of their medical importance since a number of human diseases result from abnormal melanocyte development, such as piebaldism, vitiligo, albinism, and abnormal growth and dispersal of melanoma. And the studies of colour pattern formation, neural crest development, and the cell biology and biochemistry of melanin formation and distribution in animals are also performed [13].

At present, over 100 mutations associated with albinism have been identified and described in mice and humans (see albinism database: http://www.ifpcs.org/albinism/). Colour mutations are also a common phenomenon in fish, especially in tropical ornamental fish, such as in group of Cichlid fish [7], paradise fish (*Macropodus percularis*), swordtail (*Xiphophorus helleri*) and goldfish (*Carassius auratus*) [14]. Albinism has also been observed in medaka (*Oryzias zatipes*), as well as in grass carp (*Ctenopharyngodon idella*) [15], rainbow trout *(Oncorhynchus mykiss*) [16], Japanese flounder (*Paralichthys olicaceus*) [17], black scabbard-fish (*Aphanopus carbo*) [9] and the channel catfish [18]. In most of these fishes, albinism is inherited either in an autosomal recessive manner or in an incomplete dominance manner [15, 19, 20].

Due to its biological and medical significance, the molecular mechanism of melanin biosynthesis has been extensively studied and the conserved melanin formation pathway has been elucidated in vertebrates [21–23]. Melanin is produced in the melanosome of melanocytes, which originate from melanoblast precursors. These precursor cells were developed from the dorsal neural tube during embryonic development, and migrate to their targeted destinations along defined pathways where they mature into functional melanocytes [1]. These processes are strictly controlled by many genes, among which tyrosinase (TYR) is the most important melanogenesis-related enzyme involved in the tyrosine metabolism pathway. Tyrosinase can catalyse the conversion of L-tyrosine to L-DOPA and regulate both the speed and specificity of melanogenesis [24]. In addition, tyrosinase-related protein 1 (TYRP1) and dopachrome tautomerase (DCT or TYRP2) are also important enzymes in melanin synthesis [25] and mainly regulate the eumelanin pathway. Recently, the *slc7a11* (encoding solute carrier family 7 member 11) gene has been reported to be a critical genetic regulator for pheomelanin synthesis in hair and melanocytes [26].

Pigmentation in coat colour mutations have been studied over 100 years, while most studies focused on the mammals and birds [3, 6, 27]. Limited studies were reported about fish colour mutations, of which most reports were about colour gene cloning and expression. In recent years, there were sporadic reports of genomic analysis, which are performed to determine the complete set of duplicated pigmentation genes in fish to better understand how pigment synthesis pathways have been affected by the FSGD (fish specific whole-genome duplication) [27]. Xu *et al*. not only presented a draft genome of domesticated common carp, but also performed transcriptomic analyses between two distinct domesticated strains with body colour mutation (Songpu and Hebao), which provided valuable resources for genetic breeding and molecular studies of pigmentation formation in common carp [26]. Wang *et al*. also compared the skin transcriptome between two oujiang color varieties of common carp [28]. More studies of different fish species with colour mutations are needed to better understand the molecular mechanism underlying the pigmentation in teleosts.

**Fig 1:**
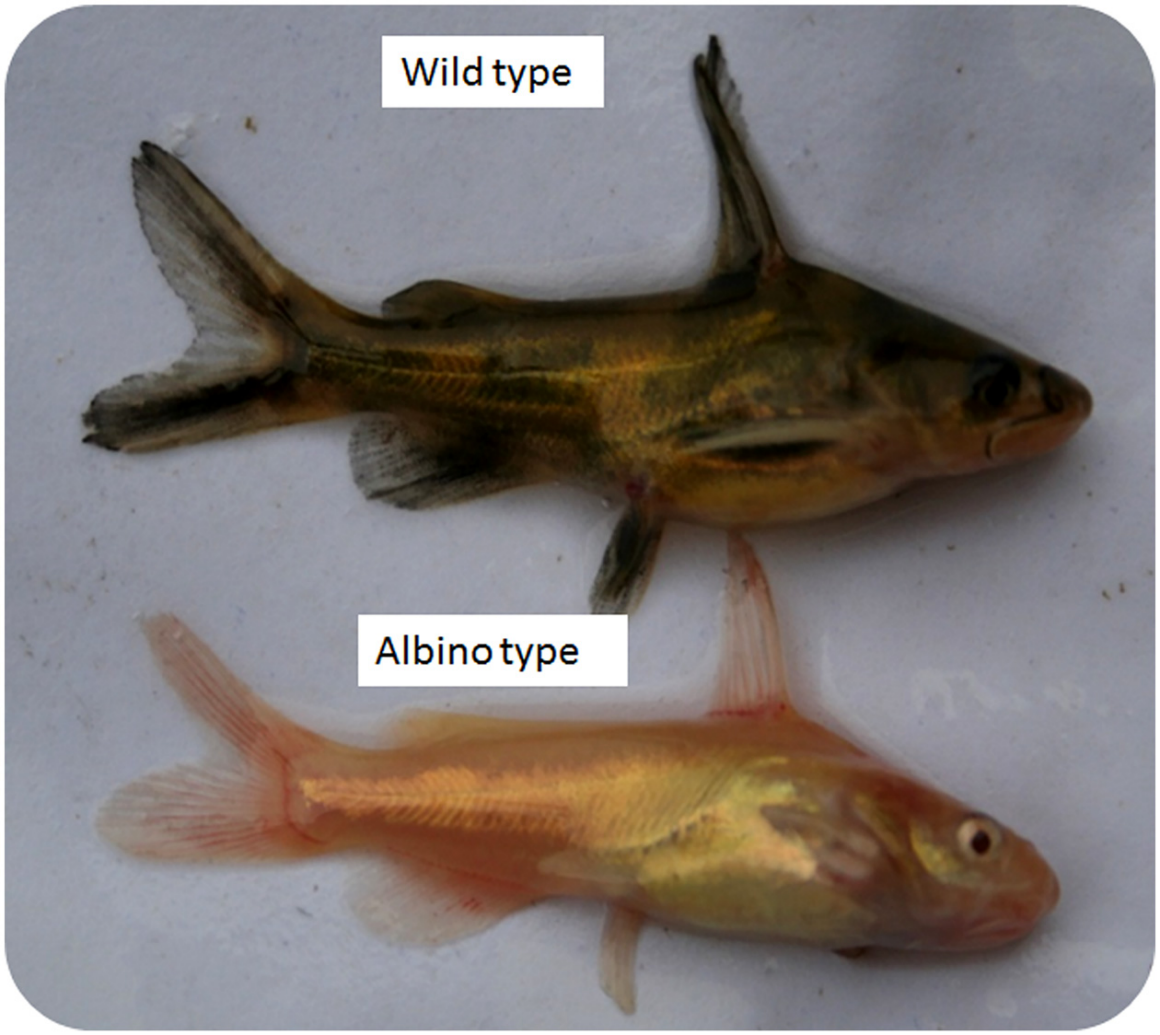
Wild and albino yellow catfish.

Yellow catfish, a teleost belonging to the family Bagridae, is one of the most important economic freshwater species in China due to its excellent meat quality. This species is extensively cultured in East and South Asia in semi-intensive and intensive systems [29]. In 2012, total production of yellow catfish reached 256,650 tonnes in China [30]. During the breeding of yellow catfish, we found a small number of fish with golden-pink eyes and golden colour all over the body (Fig 1). We consider this colour variation to result from an absence of melanin pigmentation, just like albinism reported in other species. Given the complexity of colour variation and albinism, an understanding of how melanin pigmentation is regulated has a long way to go. Transcriptome analysis of yellow catfish with golden colour will help us to better understand the mechanism for pigment biosynthesis. In this study, we sequenced the transcriptome of the normal and the newly discovered albino yellow catfish strain (thereafter called wild type and albino type, respectively) using Illumina sequencing technology. We report the differentially expressed genes (DEs) and mutations that may contribute to the phenotypic differences between these two strains, which may provide genetic information for fish colour variation.

## Materials and Methods

### Ethics statements

This study was approved by the Institutional Animal Care and Use Committees (IACUC) of Huazhong Agricultural University. No specific permits were required for the field studies described here. The study area is not privately-owned or protected in any way, and the field studies did not involve endangered or protected species.

### 2.1 Sampling

Wild type and albino yellow catfish for one year old (30 individuals for each type, ~20g each) were collected from a fish pond in Jingzhou, Hubei Province, and were transported to Huazhong Agricultural University. The yellow catfish were acclimated in an indoor tank with sufficient oxygen and fed daily at 9:00 am for 2 weeks before the experiment.

### 2.2 RNA extraction, cDNA library creation and Illumina sequencing

In order to identify potential genes subject to single-base substitution mutation and differential expression that may result in different phenotypes between wild types and albinos, we extracted and sequenced the transcriptome of both strains. For each type, we extracted and mixed equal quantity of total RNA extracted from anal fins from two individuals, and the same procedure was repeated one more time but using ten individuals instead to increase the reliability of the data. Because sequence data from public databases for *P*. *fulvidraco* is scarce, we extracted and mixed equal quantities of total RNA from skin, liver, spleen, kidney, head-kidney, and muscle of one individual for each strain to obtain coding sequences for this species. All individuals were euthanized with 300 mg/L tricaine methanesulfonate (MS 222) before dissection and total RNA was extracted using Trizol reagent (Invitrogen, USA) according to the manufacturer’s instructions. The RNA purity and integrity were evaluated by the ratio of OD_260_/OD_280_ and RIN value, respectively. RNA samples with OD_260_/OD_280_ ratio > 1.7 and RIN value > 7.5 were selected for deep sequencing. For transcriptome sequencing using Illumina’s sequencing technology, six cDNA libraries with insert sizes of 300–500 base pairs (bp) were prepared (Table 1). All libraries were sequenced using Illumina (San Diego, CA, USA) HiSeq 2000 platforms according to the manufacturer’s protocols with a read length of 100 bp. It should be noted that libraries of mixed total RNA from anal fins of ten individuals for each type (samples 5 and 6 in Table 1) were sequenced twice, once as paired-end sequence and once as single-end.

**Table 1:** Profiles of each library for the two strains subject to deep sequencing.

Sample	Strains	Number of individuals	Tissue	Reads type	Number of clean reads1	Number of clean reads2	SRA accession number
1	wild	1	skin, liver, spleen, kidney, head-kidney, and muscle	paired-end	23,206,406	23,006,146	SRR1630913
2	albino	1	skin, liver, spleen, kidney, head-kidney, and muscle	paired-end	19,813,299	19,632,145	SRR1630912
3	wild	2	anal fin	paired-end	19,920,426	19,732,153	SRR1630911
4	albino	2	anal fin	paired-end	24,111,886	23,910,559	SRR1630910
5	wild	10	anal fin	paired-end	25,564,940	25,462,585	SRR1630906
				single-end	23,880,719	-	SRR1630902
6	albino	10	anal fin	paired-end	22,479,475	22,388,185	SRR1630909
				single-end	19,996,564	-	SRR1630908

Clean reads are clipped and trimmed reads.

### 2.3 Data filtering, de novo assembly and annotation

Quality controls for original reads of each library were mainly processed using the fastx-toolkit (http://hannonlab.cshl.edu/fastx_toolkit/). First, sequencing adapters were clipped using fastx_clipper. Then fastq_quality_trimmer was used to trim the remaining data and clipped reads < 20 bp or nucleotides with phred quality scores lower than 20. Subsequently, fastx_quality_stats was used to summarize each library and GALAXY (http://galaxyproject.org/) was used to draw quality score boxplots and nucleotide distribution charts to evaluate the sequencing quality.

We combined all reads1 and reads2 separately and assembled them into contigs using Trinity software [31]. To reduce redundancy and to preserve as many coding sequences as possible, we extracted open reading frames for each contig using TransDecoder (http://sourceforge.net/projects/transdecoder/) and clustered identical proteins using CD-HIT [32]. The corresponding nucleotide sequences were retained to generate a non-redundant transcriptome that was used for subsequent analyses. To evaluate the assembly, we compared the non-redundant transcriptome to protein sequence data deposited in UniProt (http://www.uniprot.org/) using BLASTX [33]. We employed an E value of 10^−20^, and calculated the coverage between the transcript and its top hit protein. The analyses were performed for Swiss-Prot and TrEMBL, separately.

Functional annotations of the assembled sequences were achieved following the SFG pipeline [34]. Briefly, each sequence of the non-redundant transcriptome was first translated into amino acids in six frames and searched against GenBank's non-redundant protein database (NR), UniProt Swiss-Prot, and TrEMBL protein databases using local BLASTX with an E value of 10^−4^. The proteins with the highest sequence similarity were retrieved and annotated to each contig. Gene ontology (GO) terms [35] were obtained after parsing the corresponding UniProt flat files for each contig, and WEGO software [36] was then used to produce a GO functional classification for all the contigs to interpret the distribution of the species’ gene functions.

### 2.4 Differential expression analysis

Paired-end reads generated from mixed total RNA of anal fin for each sample (samples 3, 4, 5, and 6 in Table 1) were used to identify genes probably subject to differential expression between wild types and albinos. For each sample, paired-end reads were mapped back to the non-redundant transcriptome using bowtie [37]. The program rsem-calculate-expression within the software package RSEM [38] was used to estimate the expression levels for each sequence using the expectation—maximization (EM) algorithm. The expression profiles generated for each sample were combined and were used to detect differentially expressed genes between the two strains using edgeR [39] and EBSeq [40]. For edgeR, only those contigs with at least ten counts across samples were tested for diff-expression. For both tests, contigs with fold change value > 2 and the adjusted P value < 0.05 according to the False Discovery rate method of Benjamini and Hochberg [41] were selected as differentially expressed candidates.

### 2.5 Mutation sites analysis

All reads generated by this work were used to identify mutation sites by comparing the albino strain with the wild type strain. For each sample, all reads were mapped back to the non-redundant transcriptome using bwa (-n 0.005-k 5) [42], and all duplicate reads were removed using the MarkDuplicates program from the software package Picard (http://broadinstittute.github.io/picard). Then we realigned the alignments around indels using the Genome Analysis Toolkit (GATK, https://www.broadinstitute.org/gatk), and reads with minimum mapping quality < 20 were removed using samtools [43]. The processed alignments for all samples were combined using samtools and were used to generate a synchronized file using the program mpileup2sync included in the software package PoPoolation2 [44]. The synchronized file listed allele frequencies for every population at every base in the non-redundant transcriptome in a concise format. Sites with base frequencies more than five in every wild type samples but absent in every albino samples were identified as albefaction-related mutations and were compared with the predicted transcript structure to decide if they were located in five prime UTR (untranslated region), three prime UTR, or coding regions.

### 2.6 Pathway enrichment analysis

Contigs with mutation sites that changed the encoded amino acids or were identified as differentially expressed candidates were separately subject to pathway enrichment analysis using KOBAS2.0 [45] with *Danio rerio* (zebrafish) as the reference species.

### 2.7 Colour genes analysis

Using the reciprocal best blast hit method (RBB), orthologs between *D*. *rerio* and *P*. *fulvidraco* were identified. Briefly, the proteome of *D*. *rerio* was downloaded from Ensembl (http://www.ensembl.org/index.html, version 75), and RBB was performed between proteomes of the two species using blastp with the E value set to 1e-9. We also required that ortholog pairs overlapped more than 100 amino acids and more than 60% of them were identical. After that, colour-related genes in *P*. *fulvidraco* were extracted according to gene names (http://www.espcr.org/micemut/) and patterns of their expressions and mutations were scrutinized.

### 2.8 Experimental verification

In order to validate the RNA-Seq results, quantitative realtime PCR (qRT-PCR) was performed on the Roche LightCycler 480 Real-Time PCR System with three replicates using the SYBR Green PCR kit (Roche). The samples used for qRT-PCR were different from the samples used for RNA-Seq, and were collected from the anal fins of five new individuals for each strain. The gene-specific primers were designed based on the differentially expressed genes. The β-actin was used as an internal reference. The relative expression levels of DE genes were normorlized to β-actin (2^-ΔΔCt^), and all data were expressed as means±SEM. Statistical analysis was performed using GraphPad Prism 5.0 (unpaired t test). The gene and primer information is shown in Table 2.

**Table 2:** List of primers for the qRT-PCR validation of DEs identified by RNA-Seq.

Gene	Primers	sequences(5'to3')	Annealing temperature(°C)	Product Size(bps)	Contig name
*tyr*	Tyr-1278-F	CTACATGGCACCCTTCATACC	60	93	comp71391_c1_seq1
	Tyr-1370-R	AGGGTCTTGAAGGTAGGAGTA			
*ednrbla*	ednrbla-F1	CTGTGGAAGTAACCCTCATCTG	58	166	comp76216_c0_seq17
	ednrbla-R1	CAGCCACCAGTCCTTTATATCC			
*tyrp1*	TYRP1-RT-F2	GAATCCAGCAGGGAACGTGA	58	145	comp70296_c0_seq1
	TYRP1-RT-R2	GCCCTCGATGGTGTTTCTGA			
*slc7a11*	slc7a11-RT-F1	GGAATCTTCATCGCACCC	58	115	comp77711_c1_seq25
	slc7a11-RT-R1	CAGCGTAGGACAAGGCAC			
Melar-5	Melar-5-RT-F1	TATGTGCTTCATGTCCCACT	58	116	comp52331_c0_seq1
	Melar-5-RT-R1	TCTCCTTGAGCGTCTTGC			
CD8a	CD8a-RT-F1	GGACAGCGGCACTTACAG	58	287	comp73354_c0_seq23
	CD8a-RT-R1	TGACCAGAAGCAGGAGCA			
GIMAP7	GIMAP7-RT-F1	GGATTGTGCTGGTGGGAAAG	58	289	comp77845_c0_seq21
	GIMAP7-RT-R1	TTGGTGAATCTTCCGGGTTGT			
Utrophin	Utrophin-RT-F1	GTGCAACTTTCGTCCACCAC	58	266	comp80248_c1_seq8
	Utrophin-RT-R1	AGGGAATGGTAGAGCTCGGT			
Lipoprotein	Lipop-R-RT-F1	ATGGTGGATTGCGTCATA	58	249	comp71875_c1_seq1
receptor	Lipop-R-RT-R1	TCACTTGGTTCCCTTCTT			
ETFS	EFTS-RT-F1	CAAGACTACGGGCTACAC	58	204	comp69376_c0_seq2
	EFTS-RT-R1	TTCACCTCCACCATAACA			
CD99	CD99-RT-F1	CAAGACTACGGGCTACAC	58	212	comp75592_c3_seq4
	CD99-RT-R1	TCTCACAGTTCACCTCCA			
HSP70	HSP70-RT-F1	AAACTGGCTGTATGAGGAT	58	101	comp76788_c1_seq2
	HSP70-RT-R1	TGTATCGGTCTTGAATGG			
IRF-7	IRF7-RT-F1	CTCAGGCATCTACGGCTA	58	114	comp80561_c1_seq7
	IRF7-RT-R1	AGTTCATTCTGGCTTTGG			
MYCBP2	MYCBP2-F1	CATCAGGAACCGAAAGGAGAA	58	150	comp81235_c1_seq1
	MYCBP2-R1	TGGAGACCAGGCATTGTAATAG			
comp73484_c0_seq4	LOC-RT-F1	GCACAGGCGTCTTTCTCAAC	58	121	comp73484_c0_seq4
	LOC-RT-R1	AGTCTCAGCAATAGCAGCCG			
Neoverrucotoxin	Neo-RT-F1	TGGAGACCAGGCCTGTGATA	58	154	comp73484_c0_seq5
	Neo-RT-R1	ATCCCCCGTCATGTCTCTGA			
TRIMP-29	TRIMP-RT-F2	TGCTGCTCCGATTCTTGTCC	58	188	comp73484_c0_seq10
	TRIMP-RT-R2	GCTTAGGTCCAGTTGCGACA			
comp73484_c0_seq7	comp–RT-F2	GAGGGTTGGCAGTCCATTCA	58	159	comp73484_c0_seq7
	comp–RT-R2	GGACCATACGCGAACAGGAA			
Calpastatin	Calpas-l-RT-F2	ACGACTCACTTCCTCCCG	58	237	comp73354_c0_seq5
	Calpas-l-RT-R2	CCAGTTGGTCCATTACCG			
Ptprc	Ptprc-RT-F1	GACAGTGCGGACACAGAGAA	58	287	comp81041_c0_seq19
	Ptprc-RT-R1	GCTCCCTCTTCAGGCGATAC			

## Results

### 3.1 Sequencing, assembly and annotation

**Fig 2:**
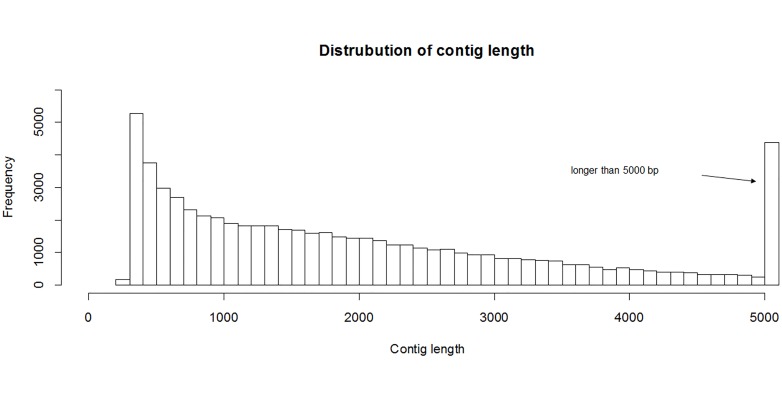
Contig length distribution of the *P*. *fulvidraco* transcriptome.

The number of clean reads (clipped and trimmed reads) obtained was > 20 M from most libraries (Table 1). Integrating these data and using a *de novo* assembly algorithm, we obtained 310,143 contigs > 200 bp. According to the TransDecoder predictions, there were 106,201 contigs encoding 144,487 proteins longer than 100 amino acids. Identical predicted protein sequences were clustered and the corresponding nucleotide sequences were extracted to form a non-redundant transcriptome, which included 64,398 sequences with the total length reached more than 135 Mb. The lengths of most contigs were < 1,000 bp, but there were also many contigs > 5,000 bp (Fig 2). The N50 contig length, median contig length, and average contig length of the non-redundant transcriptome were 3,087 bp, 1,604 bp and 2,098 bp, respectively.

**Fig 3:**
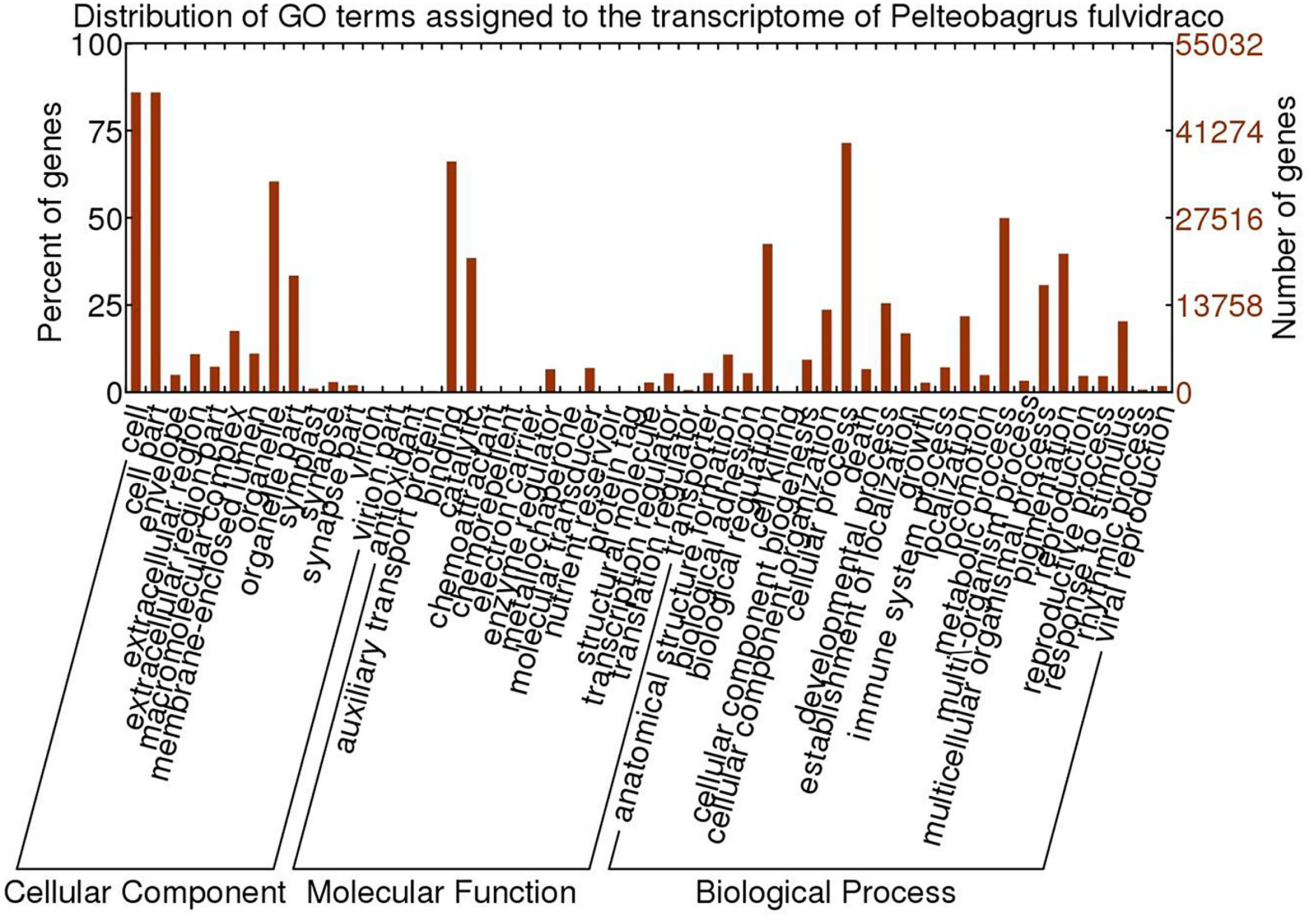
The distribution of GO terms assigned to the *P*. *fulvidraco* transcriptome.

By comparing with protein sequences deposited in NR, Swiss-Prot and TrEMBL, we annotated 61,184 (95%), 54,495(84.6%), and 59,314 (92.1%) sequences within the non-redundant transcriptome of the yellow catfish, respectively. Table 3 shows summary of the coverage of predicted proteins overlapping the homologous proteins deposit in Swiss-Prot and TrEMBL. GO terms were assigned to 55,032 sequences according to their homologs annotated by UniProt. Within these sequences, about 89.6% (49,318) were assigned to cellular component terms, 82.6% (45,473) were assigned to molecular function terms, and 80% (44,003) were assigned to biological process terms. Most represented GO terms in cellular components, molecular functions and biological processes were “cell” and “cell part”, “binding” and “cellular process”, respectively (Fig 3).

**Table 3:** The coverage of predicted proteins compared with the best hit homologs available in UniProt Swiss-Prot and TrEMBL.

Coverage (%)	Number of proteins deposit in Swiss-Prot	Number of proteins deposit in TrEMBL
90 ~ 100	8,660	13,078
80 ~ 90	2,049	2,442
70 ~ 80	1,504	2,024
60 ~ 70	1,359	2,105
50 ~ 60	1,393	2,182
40 ~ 50	1,415	2,297
30 ~ 40	1,536	2,329
20 ~30	1,629	2,378
10 ~ 20	1,299	1,828
0 ~ 10	0	0

### 3.2 Differentially expressed genes (thereafter called DEs)

Using edgeR, we detected 27 genes that were probably DEs between the wild type and albino strains (|logFC| > 1 and adjusted P value < 0.05), of which 17 (63%) were up-regulated in wild types and 10 (37%) were up-regulated in albino types. While using EBSeq, the number of DEs between wild types and albinos reached 813 (PostFC < 0.5 or > 2 and adjusted PPDE < 0.05), of which 286 (35.2%) were up-regulated in wild types and the remaining 527 (64.8%) were up-regulated in albino types. All contigs detected by edgeR except for contig comp72223_c2_seq1, which was annotated as “high mobility group protein B2”, were also detected by EBSeq. Summaries of the DEs are shown in S1 and S2 Tables compares results generated by edgeR and EBSeq for all DEs identified in this report. Summaries of DEs with adjusted P values less than 0.001 according to both of the two methods, which should be regarded as definitely DEs, are shown in Table 4. According to the pathway enrichment analyses, the DEs identified by EBSeq may participate in a number of KEGG pathways such as “Mismatch repair”, “ECM-receptor interaction”, etc (S3 Table).

**Table 4:** Summaries of differentially expressed genes (adjusted P value or PPDE < 0.001) between wild and albino types according to both edgeR and EBSeq.

ContigName	PPDE	PostFC	FDR1	logFC	PValue	FDR2	Uniprotmatch	evalue	Description
comp80248_c1_seq8	1	0.001454	0	-12.689	2.47E-11	1.15E-06	P46939	0	Utrophin
comp73484_c0_seq4	1	678.8816	0	12.60986	7.67E-11	1.32E-06	E7FBQ2	7.00E-110	Uncharacterized protein
comp73484_c0_seq5	1	622.9236	0	12.48535	8.46E-11	1.32E-06	A0ZSK3	2.00E-39	Neoverrucotoxin subunit alpha
comp73354_c0_seq38	1	0.001667	0	-12.4278	5.90E-10	6.88E-06	C6KE06	2.00E-58	CD8 alpha
comp73484_c0_seq10	1	444.998	0	11.99936	2.38E-09	2.22E-05	Q8R2Q0	9.00E-07	Tripartite motif-containing protein 29
comp73484_c0_seq7	1	538.12	0	11.93033	4.23E-09	3.29E-05	E7FBQ2	2.00E-136	Uncharacterized protein
comp73354_c0_seq43	1	627.7454	0	12.49547	1.30E-08	8.68E-05	Q2HYK0	2.00E-69	Calpastatin-like protein
comp73354_c0_seq23	1	0.0026	0	-11.7952	1.61E-08	9.40E-05	C6KE06	2.00E-58	CD8 alpha
comp81235_c1_seq1	1	388.2891	0	11.801	1.19E-07	0.000615	O75592	0	E3 ubiquitin-protein ligase MYCBP2
comp71875_c1_seq1	1	0.002816	0	-11.6659	1.99E-07	0.000821	Q28832	0	Low-density lipoprotein receptor
comp73354_c0_seq5	1	271.2564	0	9.40446	2.09E-07	0.000821	Q2HYK0	3.00E-69	Calpastatin-like protein
comp81041_c0_seq19	1	356.9776	0	11.67911	2.11E-07	0.000821	Q9IBD8	0	Receptor-type tyrosine-protein phosphatase C
comp77845_c0_seq21	1	0.002246	0	-11.5598	2.51E-07	0.000902	Q8NHV1	5.00E-34	GTPase IMAP family member 7

PPDE posterior probability of differential expression

FC fold change

FDR1 adjusted P value

FDR2 adjusted PPDE

### 3.3 Mutation analyses

We detected 806 sites resident in 616 genes that possessed one or more nucleotides present in all wild types but absent in all albinos. That is to say, we assume that single-base substitution mutations at these sites may disable the normal function of the gene and result in albinism. However, none of these sites was homozygote in all samples of the wild type; summaries of these sites are shown in S4 Table. According to the analytical predictions, 572 (71%) mutation sites within 455 genes were probably located in coding regions, and 70 (8.7%) mutation sites within 54 genes were probably located in the five prime UTR, with 255 (31.6%) mutation sites being located within 203 genes that were probably located in three prime UTR. Amino acids were changed by 129 single-base substitution mutations that were possibly located in coding regions. The results of pathway enrichment analyses for the genes where these sites reside in are shown in S3 Table, and the most represented GO terms assigned to these genes were “integral component of membrane”, “cytoplasm” and “protein binding” (S5 Table). Interestingly, we found one of the differentially expressed candidates between normal and albino fishes (serial number: comp81166_c0_seq1, FC > 15, S2 Table), which was annotated as “MHC class I antigen”, harboring one nonsynonymous site in the coding region according to the mutation analyses. We also detected 2 deletions in 2 contigs that were homozygote in albino types, but they were both located in the three prime UTR, which will not result in a frameshift or premature stop codons that may disable the protein.

### 3.4 Colour-related genes and experimental verification

We assigned *D*. *rerio* genes as orthologs for 11,978 *P*. *fulvidraco* contigs using the RBB method, within which 140 contigs were identified as differentially expressed genes using edgeR and/or EBSeq (S2 Table). However, this number should be increased because of the long time divergence between the orders Cypriniformes and Siluriformes (about 250 Mya) [46] and the stringent parameters we used for identifying orthologs. Five colour genes, namely *tyr*, *tyrp1*, *slc7a11*, *MREG*, and *ednrb1a*, were identified as up-regulated candidates in albinos according to EBSeq. According to edgeR, the expression pattern was similar, but the adjusted P value was not significant. Mutations of these genes will result in disorders of pigmentation such as albinism (http://www.espcr.org/micemut/). Quantitative RT-PCR experiments were performed to confirm the expression patterns of the four color-related genes since their up-regulation in albino strains compared to wild strains is a bit counter-intuitive. Four genes, *tyr*, *tyrp1*, *slc7a11* and *ednrb1a*, were successfully amplified and their expression patterns were in accord with the results obtained using high-throughput data. Moreover, no single-base substitution mutations were found in colour-related genes.

**Fig 4:**
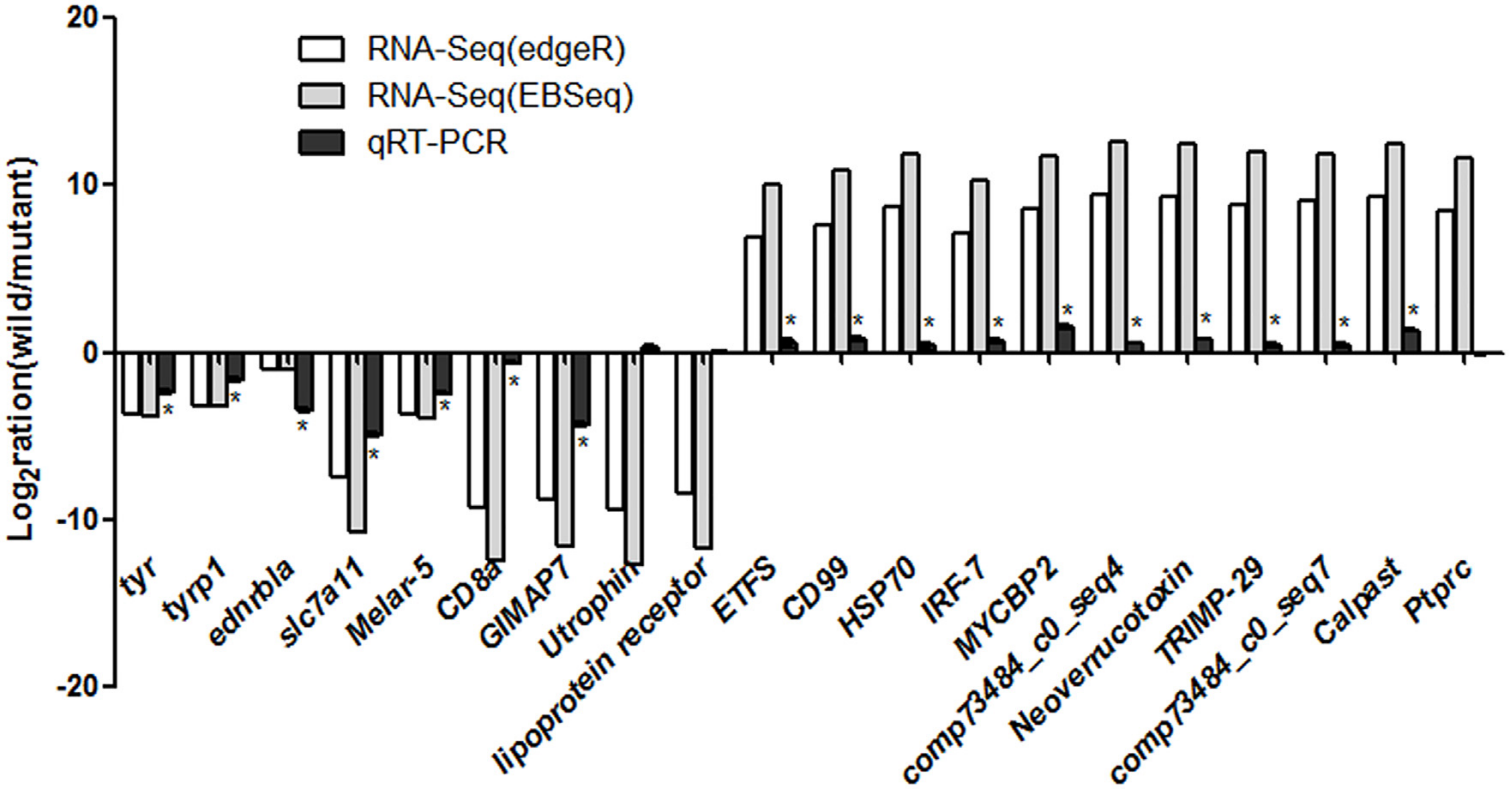
The results of qRT-PCR for the DEs identified by RNA-Seq and subject to validation.

To further validate the DE genes detected by RNA-Seq, another sixteen candidates (twelve were from Table 4 and another 4 were randomly selected from S2 Table) were selected and qRT-PCR amplification confirmed the expression patterns of 13 genes. For the other three DE candidates (lipoprotein receptor, Utrophin and Ptprc), no significant difference was detected (Fig 4).

## Discussion

In this report, a transcriptome represented most genes expressed in *P*. *fulvidraco* was generated and annotated based on the sequence homology to proteins deposit in public databases. The coverage of predicted proteins showed that 14,965 (23.2%) sequences overlapping for more than 50% of the length of homologous proteins deposit in Swiss-Prot, of which 10,709 (16.6%) sequences covered more than 80% (Table 3). Compared with homologous proteins in TrEMBL, the number of sequences overlapping for more than 50% of their length reached 21,831 (33.9%), of which 15,520 (24.1%) sequences overlapped more than 80%. Given the very strict parameter used (E value = 1e-20) when searching for homologs, the statistics presented above are expected to be conservative. Nonetheless, these statistics suggest that we have generated a high quality transcriptome for *P*. *fulvidraco*. A portion of contigs without any hits may represent lineage-specific genes and may partially explain the uniqueness of *P*. *fulvidraco*. Compared to other RNA-Seq reports, the ratio of annotated contigs was moderately high [47]: this is because we removed all contigs except those encoding more than 100 amino acids.

Based on the *de novo* assembled transcriptome, a number of differentially expressed genes were identified. Elsewhere, Seyednasrollah *et al*. compared eight packages detecting DEs based on real data and claimed that the number of detected DEs increased when the number of replicate samples was increased for most packages, including edgeR and EBSeq [48]. Specifically, edgeR showed a large range of variability when the number of replicates was very small, while EBSeq may be too liberal. As a result, EBSeq detected many more DEs than edgeR when using only two replicate samples; increasing the number of replicates is expected to narrow the gap. Simulation and real data showed that most genuine DEs ranked at the top when sorting their significance such as nominal P value from the smallest to the largest [48, 49]. This method is not affected by the direction of differential expression and edgeR performed better than EBSeq when the sample size was very small. Correspondingly, based on the results obtained by edgeR and EBSeq, most DEs identified in this report (list in S2 Table) ranked in the top 2,000 in order of their significance.

**Fig 5:**
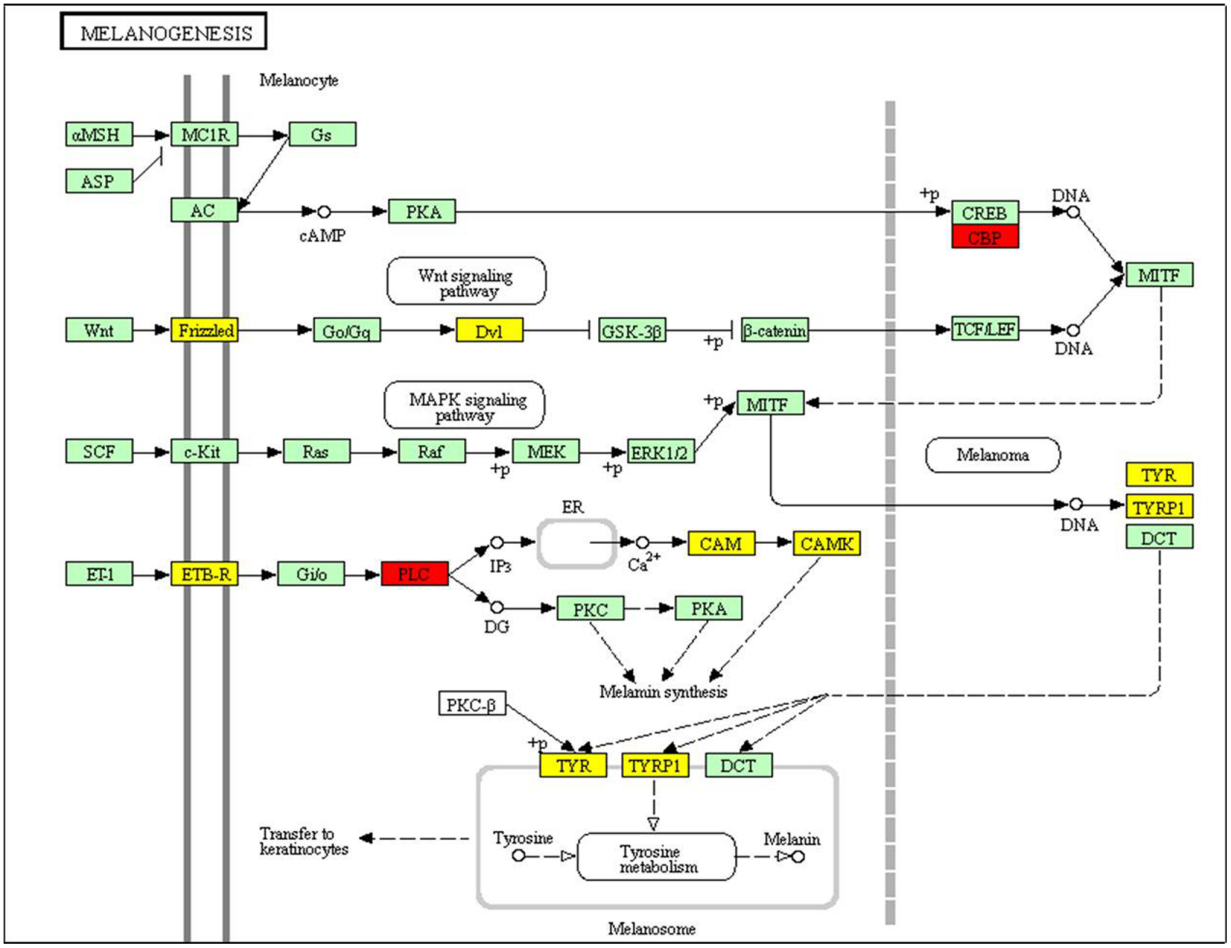
Differentially expressed genes between wild and albino types in anal fins and their involvement in the melanogenesis pathway. Genes marked in red are up-regulated and in yellow represented genes are down-regulated in wild type versus in albino type, respectively. Comparison between RNA-Seq results (identified by both edgeR and EBSeq) and qRT-PCR validation results. X-axis shows genes validated in this study; Y-axis shows Log2Ratio of expression of wild versus albino types. “*” indicates a significant difference between the two strains.

Pathway enrichment analyses of differentially expressed genes identified by EBSeq (S3 Table) revealed that eleven candidates take part in the pathway “Melanogenesis” and most of these genes, including the important tyrosinase (TYR) and tyrosinase-related protein 1 (TYRP1), were up-regulated in albino types (Fig 5). This may result from the up-regulation of cystine/glutamate transporter in albino types (slc7a11, S2 Table), which transports cystine into melanocytes, switch off the eumelanin (brown to black pigment) synthesis pathway and promote the synthesis of pheomelanin (yellow to red pigment). The lack of substrates for the synthesis of eumelanin may result in the high level of relevant enzymes and corresponding transcripts and reduce the melanin synthesis, which contribute to the abnormal body colour of the albino strain. The similar results were found in common carp [26]. Moreover, KEGG pathways such as “mismatch repair”, “base excision repair”, etc., were significantly enriched in differentially expressed genes (adjusted P value < 0.05, S3 Table). These are cellular mechanisms that repair damaged DNA. We suggest that without the protection of sufficient melanin, albino fishes were much more easily subject to DNA damage caused by ultraviolet (UV). Exposure to UV will trigger intracellular repair mechanisms with the result that the expression levels of many related genes in albino types were significantly different from those in wild types.

Genes harboring mutations that may influence their normal functions in albinos were also identified. However, no pathway potentially related to different fish body colour was detected among these genes with nonsynonymous mutations (S3 Table). Thus, we conclude that phenotypic differences between wild types and albinos were mainly the result of differential expression of related genes rather than single-base substitution mutations in coding regions. This was in accordance with published analyses from the genome resequencing projects for *Saccharomyces cerevisiae* (yeast) and *Gasterosteus aculeatus* (threespine stickleback). For both it was claimed that at the beginning of divergence, alterations of expression pattern should be much more frequent than expected [50, 51], and should be even more frequent than substitutions of encoded amino acids [51].

## Conclusion

We have generated the transcriptome of the Chinese yellow catfish, *P*. *fulvidraco*, which will be important for subsequent analyses of this economically important fish. More widely, it should also be important for comparative genomics and evolutionary analyses in the Siluriformes. We conclude that the dominating molecular events underlying phenotypic difference between albino and normal *P*. *fulvidraco* individuals result from differential expression of related genes rather than single nucleotide mutations located in coding regions. Moreover, we found genes that take part in pathways “mismatch repair” and “base excision repair” that may be responses to ultraviolet exposure and result in cellular damage, which were significantly enriched in differentially expressed genes between wild types and albinos.

## Supporting Information

S1 Table Summaries of differentially expressed genes between wild and albino types identified by edgeR and EBSeq. (XLS)

S2 Table Comparisons of results generated by edgeR and EBSeq for differentially expressed genes between wild and albino types. (XLS)

S3 Table Pathway enrichment analyses for differentially expressed genes between wild and albino types identified by EBSeq and genes in which non-synonymous mutations reside. (XLS)

S4 Table Summaries of mutation sites with one kind of nucleotide present in the wild type strain but absent in the albino strain. Note that some contigs may encode more than one protein sequence according to the prediction.
(XLS)

S5 Table Single-base substitution that was possibly located in coding regions and result in non-synonymous mutations. (XLS)
